# Primary versus rerupture of the anterior cruciate ligament: rupture site patterns and graft elongation—a systematic review and meta-analysis

**DOI:** 10.1186/s43019-026-00313-9

**Published:** 2026-04-03

**Authors:** Riccardo D’Ambrosi, Beata Ciszkowska-Łysoń, Luca Maria Sconfienza, Christian Fink, Robert Śmigielski

**Affiliations:** 1 IRCCS Ospedale Galeazzi – Sant’Ambrogio, Milan, Italy; 2 Department of Biomedical Sciences for Health, University of Milan, Milan, Italy; 3 Department of Radiology, MIRAI Clinic, Otwock, Poland; 4 Department of Trauma, Orthopedics and Sports Medicine, Life Medical Center, Warsaw, Poland; 5 Gelenkpunkt-Sports and Joint Surgery FIFA Medical Centre of Excellence, Innsbruck, Austria; 6 Research Unit for Orthopaedic Sports Medicine and Injury Prevention (OSMI), Private University for Health Sciences Medical Informatics and Technology, Innsbruck, Austria; 7 Department of Descriptive and Clinical Anatomy, Center for Biostructure Research, Medical University of Warsaw, Warsaw, Poland

**Keywords:** Anterior cruciate ligament, ACL reconstruction, Graft failure, Rupture pattern, Elongation, Meta-analysis, Vascularization, Reinjury risk

## Abstract

**Background:**

While the rupture pattern of the native anterior cruciate ligament (ACL) has been well characterized—most frequently occurring in the proximal third of the ligament—there is limited consensus on whether reconstructed ACLs fail in a similar fashion. The purpose of this meta-analysis is to compare rupture localization patterns between first ruptures and reruptures.

**Materials and methods:**

MEDLINE (PubMed), Embase, and the Cochrane Library databases were searched to identify studies analyzing rupture locations after both first rupture and rerupture. The main extracted outcome was the anatomical site of ACL rupture, categorized as proximal, mid-substance, distal, or elongation. A random-effects meta-analysis was performed to calculate pooled proportions and odds ratios (ORs), with between-study heterogeneity assessed using the *I*^2^ statistic.

**Results:**

The pooled meta-analysis revealed no significant differences (*p* > 0.05) in the distribution of proximal, mid-substance, or distal rupture locations between the first rupture and rerupture groups. However, graft elongation was significantly more frequent in reruptures (*p* < 0.05). Using first rupture as the reference, the odds of elongation were 1.63-fold higher in the rerupture group (OR 1.63, 95% CI 1.46–1.83; *p* < 0.001).

**Conclusions:**

This meta-analysis found no statistically significant differences in rupture-site localization between first ruptures and reruptures, indicating that reconstructed ACLs tend to fail in anatomical patterns resembling those of the native ligament. However, the markedly higher incidence of graft elongation observed in the rerupture cohort suggests a distinct mode of failure. These findings highlight graft elongation as a specific mechanism that may be influenced by surgical or biological factors, warranting further investigation.

Level of evidence: Systematic review and meta-analysis of Level 4 studies.

*Study registration*: PROSPERO Registry CRD420251073835.

**Supplementary Information:**

The online version contains supplementary material available at 10.1186/s43019-026-00313-9.

## Introduction

Anterior cruciate ligament (ACL) tears are a prevalent and significant cause of morbidity in athletes, with an estimated annual incidence of 68.6 per 100,000 person-years in the general population and over 200 per 100,000 in high-risk athletic cohorts. ACL reconstruction (ACLR) is widely performed, with over 200,000 procedures annually in the USA alone [1–4].

Despite advances in surgical techniques and postoperative rehabilitation, graft failure remains a major clinical concern, with reported retear rates ranging from 5% to 25%, depending on age, activity level, and graft type [5–8].

Interestingly, up to 30% of patients under 25 years of age who return to pivoting sports sustain a second ACL injury, either to the reconstructed knee or to the contralateral side [9–11].

While the rupture pattern of native ACLs is well established—with most tears occurring in the proximal third—evidence remains inconsistent regarding whether reconstructed ACLs fail in similar anatomical locations. Some studies suggest that graft ruptures may occur more frequently at the femoral tunnel or intrasubstance region, potentially owing to altered biomechanics, tunnel position, or graft remodeling. Clarifying whether graft failures mirror native rupture patterns is essential to guide surgical planning and reinjury prevention [12–15]. This meta-analysis aimed to determine whether reconstructed ACLs fail in a similar anatomical pattern as native ligaments, by comparing rupture-site distribution (proximal, mid-substance, and distal) and the occurrence of graft elongation between first ruptures and reruptures.

## Materials and methods

A systematic search strategy was developed according to the Preferred Reporting Items for Systematic Reviews and Meta-Analyses (PRISMA) guidelines and registered in the PROSPERO Registry CRD420251073835 [16, 17]. The AMSTAR-2 checklist was used to confirm the quality of the systematic review [18]. The TITAN checklist was fulfilled to transparently report the use of artificial intelligence [19]. An electronic search of MEDLINE (PubMed), Embase (Elsevier), and the Cochrane Library was conducted from database inception to 24 June 2025, restricted to English-language publications. The search was first executed on June 10, 2025, and updated on June 24, 2025. The following Boolean terms were used: ‘ACL reconstruction’ OR ‘anterior cruciate ligament reconstruction’ OR ‘ACL’ AND ‘location’ OR ‘rupture’ OR ‘anatomy’ OR ‘distal’ OR ‘proximal’ OR ‘mid-substance’ OR ‘elongation’ OR ‘tibial’ OR ‘femoral’.

### Research question (PICO framework)

The research question was structured according to the PICO framework as follows [20]:

- **Population (P):** patients with ACL injury, either native or reconstructed
- **Intervention (I):** ACLR (reconstructed ACLs evaluated at the time of rerupture)
- **Comparison (C):** native ACL ruptures (first-time injuries)
- **Outcome (O):** anatomical localization of ligament rupture (proximal, mid-substance, distal) and occurrence of elongation

The formulated research question was: “Do reconstructed ACLs fail in the same anatomical location as native ACLs?”.

### Eligibility criteria

The literature included in this study was selected on the basis of the following criteria:

#### Study design

Randomized controlled trials (RCTs), controlled (nonrandomized) clinical trials (CCTs), prospective and retrospective comparative cohort studies, case‒control studies and case series were included. Case reports and case series that did not report data on the location of ACL rupture were excluded.

#### Participants and interventions

We included studies conducted on skeletally mature patients that reported the exact location of the rupture of the native or reconstructed ACL. In the case of double bundle reconstruction, the two bundles were considered separately and independently.

#### Types of outcome measures

**Fig. 1:**
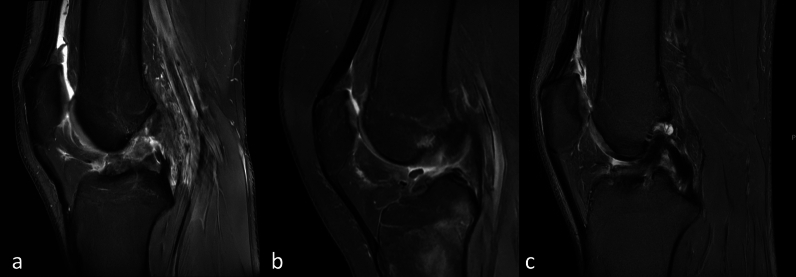
Sagittal proton density-weighted MR image of the knee demonstrating: **a** a distal rupture of the anterior cruciate ligament, **b** a mid-substance rupture of the anterior cruciate ligament, and **c** a proximal rupture of the anterior cruciate ligament

The main extracted and recorded outcome measure was the location of the ACL rupture (native or reconstructed). ACL localization was divided as follows [21–24]:

- Distal (or tibial), when affecting the lower third of the ligament near the tibial insertion (Fig. 1a)
- Mid-substance, when involving the central third (Fig. 1b)
- Proximal (or femoral), when located within the upper third of the ligament near the femoral footprint (Fig. 1c)
- Elongation: defined as a progressive increase in anterior tibial translation ≥ 3 mm compared with initial postoperative measurements, in the absence of graft rupture on clinical or imaging assessment. Although the terminology was not uniformly applied across studies, this standardized definition was adopted to harmonize data extraction and maintain consistency with previously published biomechanical and clinical literature on graft elongation [25–27].

In studies involving double-bundle ACLR, the anteromedial (AM) and posterolateral (PL) bundles were analyzed as separate observations. This approach was adopted to preserve anatomical specificity in rupture localization, since each bundle has distinct femoral and tibial footprints, tension patterns, and potential failure sites. This methodological choice is consistent with previous analyses of intra-articular rupture patterns and anatomical studies describing the functional independence of the two bundles [25–27].

Definitions of rupture and elongation followed those provided in the included studies. In general, a rupture was defined as a complete or partial discontinuity of ACL fibers confirmed by intraopeartive or magnetic resonance imaging (MRI) findings. Elongation referred to loss of graft tension or signal attenuation on MRI in the absence of fiber disruption, often associated with increased anterior laxity on clinical or instrumented testing (e.g., KT-1000, GNRB). The timing of assessment corresponded to the moment when the failure was identified—either intraoperatively during revision surgery or on MRI obtained during follow-up [22, 27–31]. The definitions and time points reported in each study are summarized in Table 1.

**Table 1:** Characteristics of the studies included in the meta-analysis

	Study design	Number of patients	MINORS	Index category	Reconstruction type	Rupture/elongation assessment method	Assessment timing	Graft type for ACLR
Van der List et al. [44]	Case series	350	12	Primary	Not available	MRI (1.5 or 3.0 T)	8 days from injury	
Hoogeslag et al. [37]	Diagnostic cohort study	28	16	Primary	Not available	MRI (1.5 T) + arthroscopic	5 days for MRI 14 days for arthroscopy	
Grøntvedt et al. [36]	Prospective randomized	147	20	Primary	Arthrotomy	Intraoperative	Within 10 days after the injury	
Cross et al. [35]	Retrospective	30	10	Primary	Arthrotomy	Intraoperative	Within 2 weeks from injury	
Liljedahl et al. [38]	Prospective	35	8	Primary	Arthrotomy	Intraoperative + arthrography	Acute (not specified)	
Ubl et al. [43]	Retrospective	158	13	Primary	Not available	MRI	3 days	
Shu et al. [41]	Prospective	34	10	Primary	Not available	MRI (3.0 T) + arthroscopic	2–3 weeks for arthroscopy	
Marshall et al. [39]	Prospective	70	9	Primary	Arthrotomy	Intraoperative	5.3 days from injury	
Tan et al. [42]	Retrospective	291	9	Primary	Not available	MRI (3.0 T)	30 days from injury	
Sgaglione et al. [45]	Retrospective	71	9	Primary	Arthrotomy	Intraoperative	9.1 days from injury	
Sherman et al. [40]	Retrospective	50	8	Primary	Arthrotomy	Intraoperative	7.1 days from injury	
Van Eck et al. [29]	Cohort study	59	13	Rerupture	Arthroscopy	Arthroscopy Footage	59 months from initial surgery to rerupture	Single-bundle 21 allograft 33 autograft
Magnussen et al. [30]	Retrospective cohort study	28	12	Rerupture	Arthroscopy	Operative report and intra-operative photo	13.4 weeks from re-injury to revision surgery	Autografts: 13 hamstrings 6 BPTB 1 Iliotibial band Allografts: 5 BPTB 5 tibialis anterioris 2 Achilles tendon
Van Eck et al. [28] (PL)*	Prospective	82	12	Rerupture	Arthroscopy	Arthroscopy Footage	18.6 months from initial surgery to rerupture	Double-bundle reconstruction
Van Eck et al. [28] (AM)*	Prospective	100	12	Rerupture	Arthroscopy	Arthroscopy Footage	18.6 months from initial surgery to rerupture	Double-bundle reconstruction

^*^Same study. The two bundles were considered separately. *PL* posterolateral, *AM* anteromedial, *BPTB* bone-patellar tendon to bone, *MRI* magnetic resonance imaging, *T* tesla

### Data collection and analysis

#### Study selection

The retrieved articles were first screened by title and, if relevant, further screened by reading the abstracts. After studies that did not meet the eligibility criteria were excluded, the entire content of the remaining articles was assessed for eligibility. To minimize the risk of bias, the reviewers independently assessed all retrieved records and discussed any discrepancies in study inclusion or exclusion. In cases of disagreement, the senior investigator made the final decision. At the end of the process, additional studies that might have been missed were searched manually by going through the reference lists of the included studies and relevant systematic reviews.

#### Data collection process

Data were extracted from the selected articles by the first two authors using a computerized tool created with Microsoft Access (version 2010, Microsoft Corp, Redmond Washington). Each article was validated again by the first author before analysis. For each study, data on the localization of the rupture were recorded.

### Level of evidence

The Oxford Levels of Evidence set by the Oxford Centre for Evidence-Based Medicine were used to categorize the level of evidence [32].

### Evaluation of the quality of studies

The quality of the selected studies was evaluated using the Methodological Index for Nonrandomized Studies (MINORS) score. The checklist includes 12 items, of which the last 4 are specific to comparative studies. Each item was given a score of 0–2 points. The ideal score was set at 16 points for noncomparative studies and 24 points for comparative studies [33].

### Statistical analysis

For each outcome, a random-effects model was applied using the DerSimonian–Laird estimator for between-study variance. Raw proportions were stabilized using the Freeman–Tukey double arcsine transformation to reduce variance instability and handle proportions close to 0 or 1, as commonly recommended in meta-analyses of proportions. Differences between first rupture and rerupture were explored using a mixed-effects meta-regression model with a common between-study variance component across groups. Overall between-study heterogeneity within each pooled model was assessed using Cochran’s *Q* test and quantified with the Higgins *I*^2^ statistic, while between-group heterogeneity was evaluated using Cochran’s *Q* test for subgroup differences. Pooled estimates are presented as proportions with corresponding 95% confidence intervals, and group comparisons are reported as odds ratios with 95% confidence intervals, using first rupture as the reference category. Statistical heterogeneity was considered substantial if *I*^2^ > 50% [34]. All tests were two-tailed, and *p* < 0.05 was considered statistically significant. Statistical analyses were performed using R (version 4.3.0; R Foundation for Statistical Computing, Vienna, Austria; https://www.R-project.org/), with the meta (version 8.0.1) and metafor (version 4.2.0) packages.

## Results

**Fig. 2:**
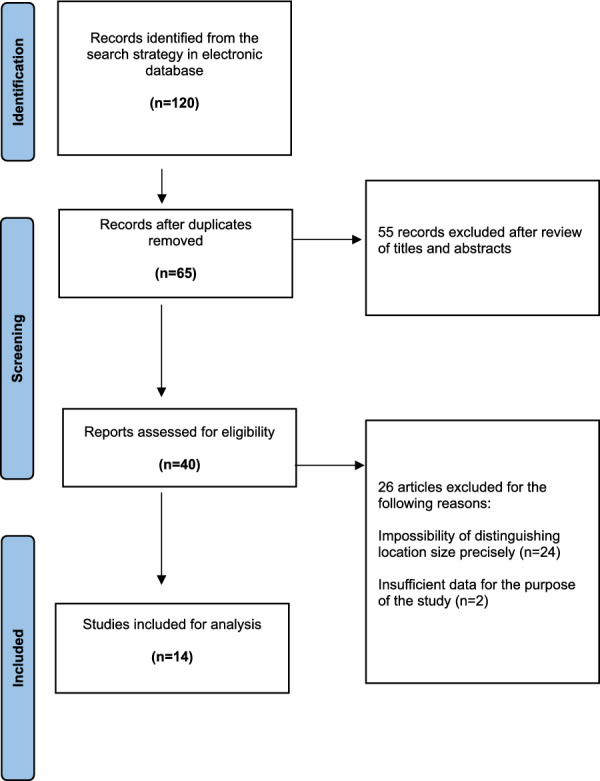
PRISMA flow chart indicating the inclusion of research articles for final analysis

A thorough search of the three electronic databases yielded 120 records. After removing 55 duplicates, 65 studies were screened by title and abstract, and 40 full-text articles were assessed for eligibility. Following full-text review, 14 studies met the inclusion criteria [28–30, 35–45], including a total of 1533 patients (11 studies on native ACL and 3 on reconstructed ACL). The PRISMA flowchart is shown in Fig. 2, and study characteristics are detailed in Table 1.

### Distal ruptures

**Fig. 3:**
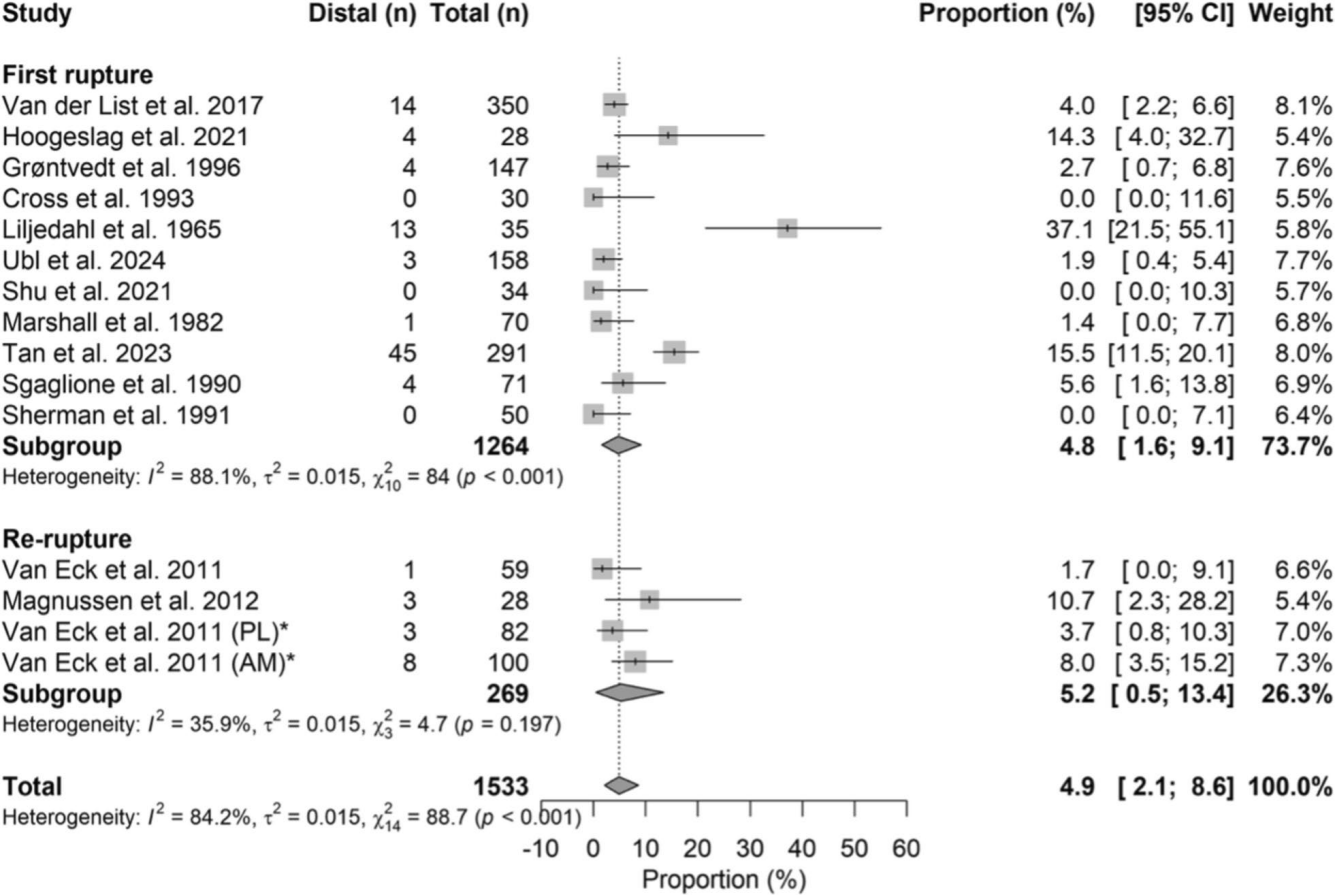
Forest plot of the distal rupture rate between the first rupture group and the rerupture group

The pooled incidence of distal rupture was 4.8% (95% CI 1.6–9.1) in first ruptures and 5.2% (95% CI 0.5–13.4) in reruptures, with no significant difference between groups (OR = 1.01, 95% CI 0.86–1.19; *p* = 0.891). Figure 3 shows the corresponding forest plot.

### Mid-substance ruptures

**Fig. 4:**
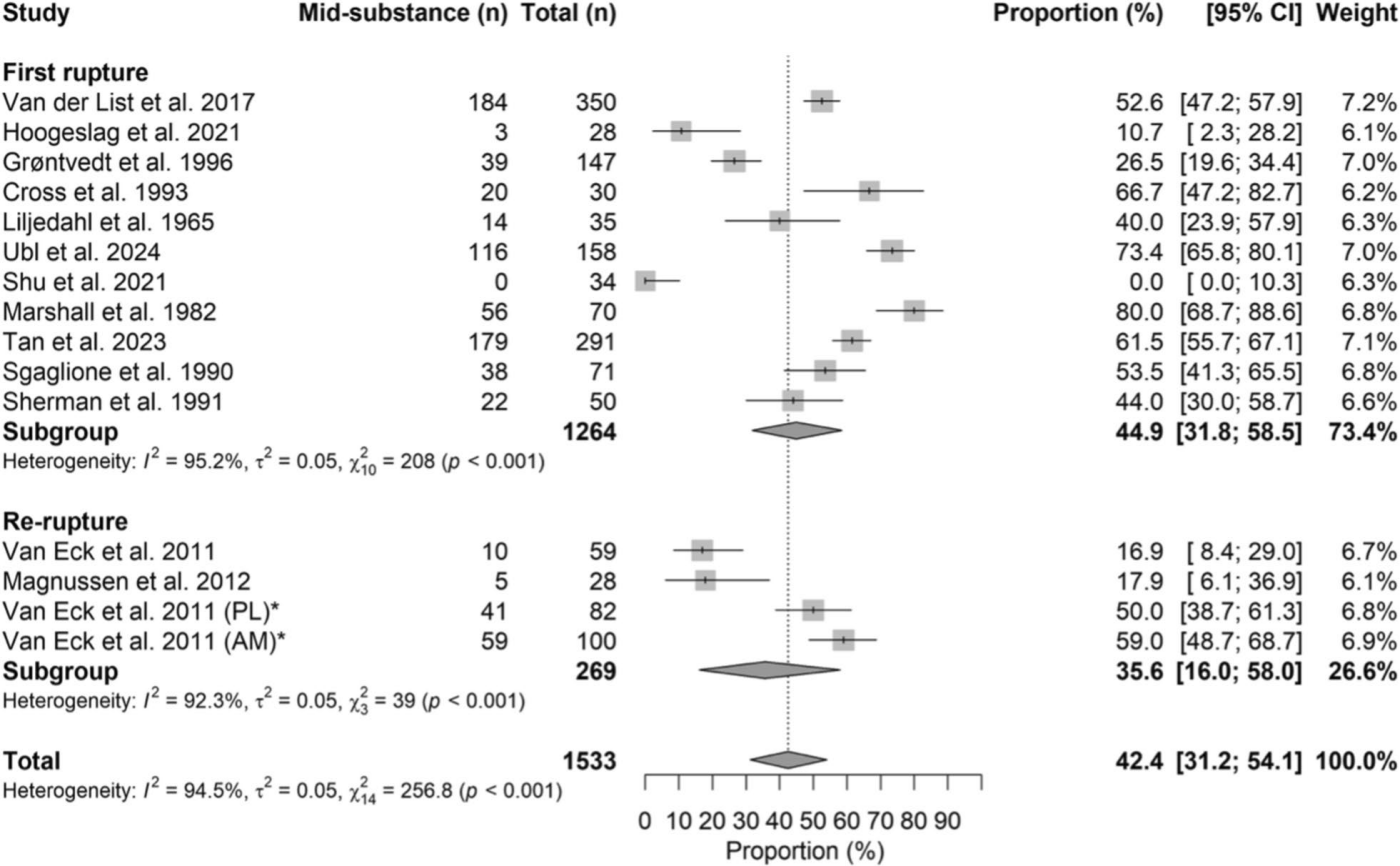
Forest plot of the mid-substance rupture rate between the first rupture and rerupture groups

Mid-substance lesions occurred in 44.9% (95% CI 31.8–58.5) of first ruptures and 35.6% (95% CI 16.0–58.0) of reruptures, without significant differences between groups (OR = 0.91, 95% CI 0.70–1.18; *p* = 0.481). The forest plot is presented in Fig. 4.

### Proximal ruptures

**Fig. 5:**
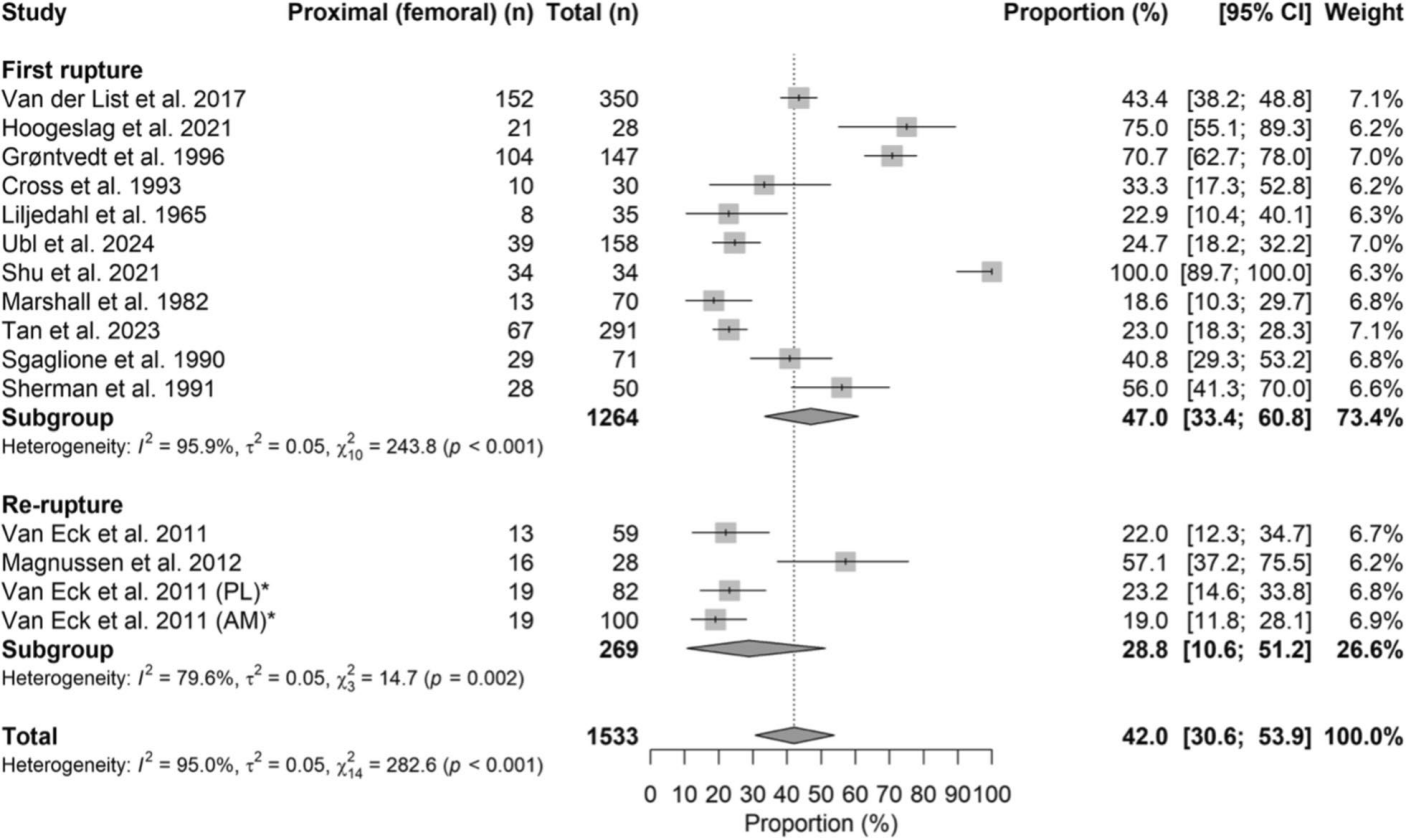
Forest plot of the proximal rupture rate between the first rupture group and the rerupture group

Proximal ruptures accounted for 47.0% (95% CI 33.4–60.8) of first ruptures and 28.8% (95% CI 10.6–51.2) of reruptures, showing no significant difference between groups (OR = 0.83, 95% CI 0.64–1.08; *p* = 0.170). Figure 5 shows the corresponding forest plot.

### Elongation

**Fig. 6:**
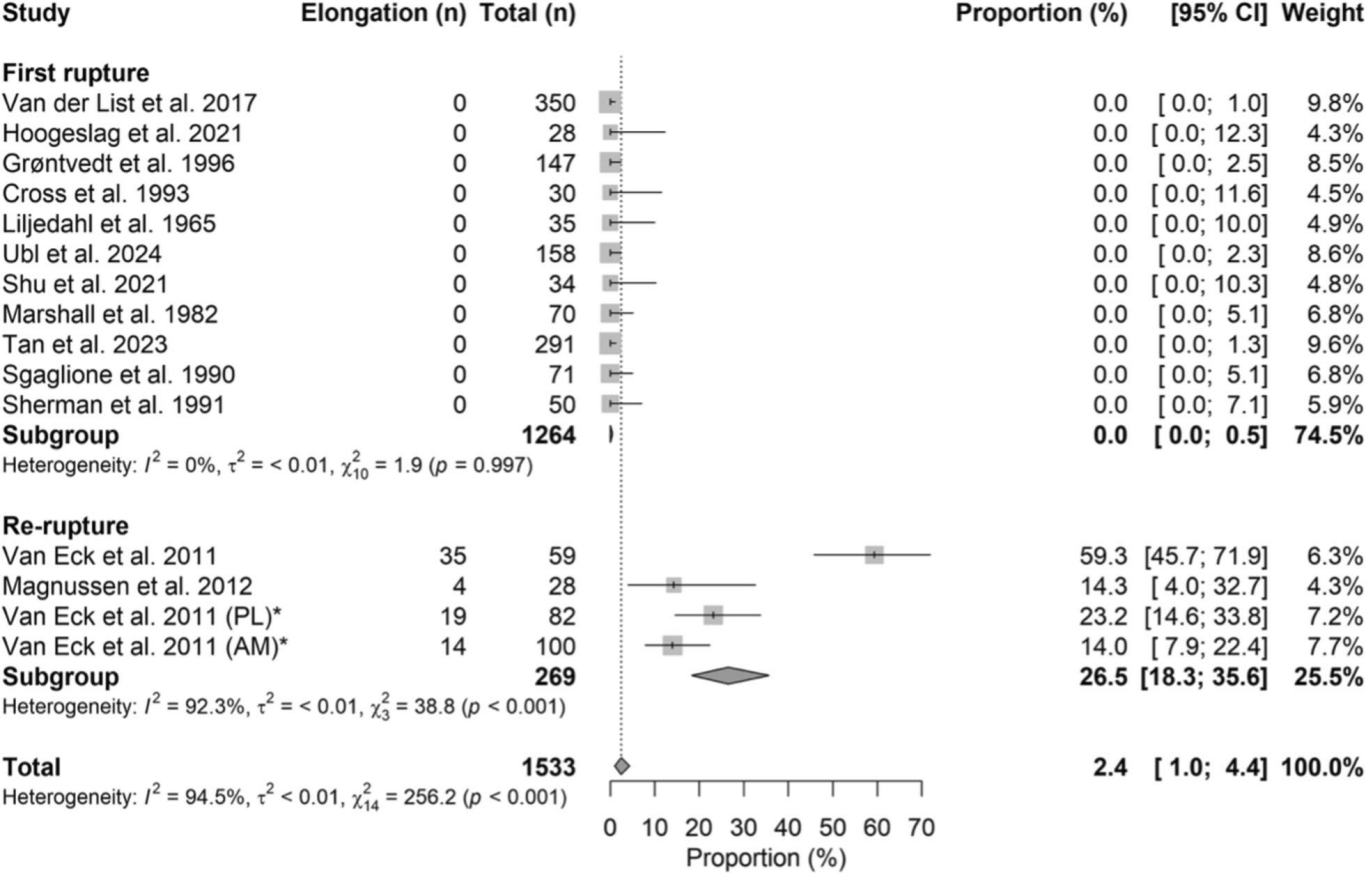
Forest plot of elongation rates between the first rupture and rerupture groups

Graft elongation occurred in 0.0% (95% CI 0.0–0.5) of first ruptures and 26.5% (95% CI 18.3–35.6) of reruptures, representing a significantly higher rate in reruptures (OR = 1.63, 95% CI 1.46–1.83; *p* < 0.001). The corresponding forest plot is shown in Fig. 6.

### Overall comparison and heterogeneity

**Fig. 7:**
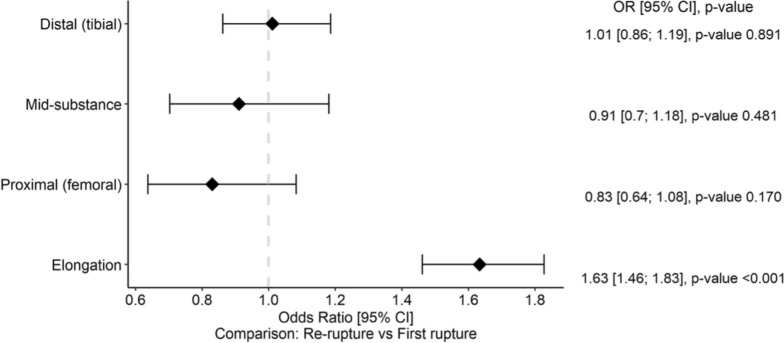
Forest plot illustrating the pooled odds ratios for rupture localization patterns between the first rupture and rerupture groups. Using the first rupture group as the reference, the rerupture group demonstrated higher odds for proximal rupture and elongation, and lower odds for mid-substance and distal ruptures (overall pooled OR = 1.63, 95% CI 1.46–1.83; *p* < 0.001)

The pooled comparison across all rupture patterns is presented in Fig. 7, illustrating the overall odds ratio distribution among distal, mid-substance, proximal, and elongation lesions. Between-study heterogeneity was substantial across most models (*I*^2^ = 84–95%), except for distal and rerupture subgroups, which showed lower heterogeneity. Detailed heterogeneity statistics (*I*^2^, *τ*^2^, *Q*-test) for each analysis are provided in Supplementary File 1.

## Discussion

This meta-analysis demonstrated that rupture-site localization was broadly comparable between native ACL tears and reruptures of reconstructed grafts, with both groups predominantly exhibiting proximal and mid-substance failure. However, a notable difference emerged: graft elongation was observed exclusively in the reconstructed ACL cohort, highlighting a distinct postoperative failure mechanism.

Native ACL tears typically occur in the proximal third of the ligament owing to anatomical and vascular factors, and our findings confirm that this pattern is largely preserved following reconstruction [46–48]. Despite differences in structure and biology, reconstructed grafts appear to experience similar mechanical constraints, which may reflect the ability of current surgical techniques to effectively replicate native ligament alignment. Although prior reports suggested more frequent femoral tunnel or tibial-side ruptures associated with tunnel malposition or altered biomechanics, our pooled analysis did not demonstrate a significant shift in rupture location [49–51].

The higher incidence of graft elongation in reruptures—occurring in 26.5% of reconstructed ACLs compared with none of the first-time ruptures—represents one of the most clinically meaningful findings. Elongation, defined as a progressive ≥ 3 mm increase in anterior tibial translation without fiber discontinuity, reflects a gradual loss of functional graft integrity rather than an acute mechanical disruption [25–27]. This pattern was consistently reported to occur later in follow-up than complete graft failures, supporting its characterization as a chronic, progressive process.

It is important to note that progressive laxity and attenuation-like changes may also occur in native ACLs, particularly in chronic partial tears, degenerative conditions, and osteoarthritic knees. Recent biomechanical work has shown that native ACL bundles in osteoarthritic joints exhibit reduced stiffness, altered viscoelastic behavior, and collagen disorganization—features consistent with functional attenuation in the absence of an acute rupture [52].

Complementing these mechanical findings, histological analyses have demonstrated that native ACLs frequently undergo aging- and osteoarthritis-related degeneration, including collagen fiber disorientation, mucoid and cystic changes, and macroscopic thinning, which may precede or progress independently of articular cartilage damage [53]*.*

Therefore, the lack of elongation events observed in first-time ruptures in our meta-analysis should not be interpreted as evidence that elongation cannot occur in native ACLs; rather, it reflects that graft elongation in reconstructed knees represents a postoperative, biologically and mechanically distinct failure pathway.

Graft elongation was typically observed at longer intervals after the index reconstruction compared with complete reruptures. Specifically, elongation occurred at a mean of 18.6 (6–39) months in the double-bundle series by Van Eck et al., 59 (5–211) months in the single-bundle cohort from the same author, and approximately 11–14 months in the mixed population analyzed by Magnussen et al. [28–30]. This temporal pattern reinforces the interpretation of elongation as a chronic, progressive process rather than an acute event. Although this standardized definition harmonized heterogeneous reporting across studies, variability in the timing and method of elongation assessment remains an inherent limitation of the available evidence [54–56].

Clinically, graft elongation may produce instability symptoms comparable to those observed in complete graft failure, even when the graft appears continuous on MRI. In such cases, relying solely on structural continuity may underestimate functional insufficiency. Recognition of elongation as a source of instability is therefore essential to ensure that treatment decisions— including the consideration of revision ACLR—are based on clinical and functional assessment rather than imaging appearance alone.

From a surgical and rehabilitation standpoint, graft elongation may develop gradually during early postoperative loading, particularly when tunnel placement or fixation orientation leads to uneven load distribution. This risk can be amplified by premature return to sport, especially in young athletes whose biological healing may lag behind mechanical demands [57–59].

The disparity between the rupture rates and elongation rates may be partly explained by differences in vascularization between native and reconstructed ACLs. The native ACL is supplied primarily by branches of the middle genicular artery, providing a robust microvascular network that supports homeostasis and repair. In contrast, reconstructed grafts—either autografts or allografts—undergo a prolonged and incomplete process of revascularization known as “ligamentization.” This process is both spatially and temporally heterogeneous, with animal studies showing persistently reduced vascular density and disorganized collagen structure even 6–12 months after surgery [60–63].

Incomplete vascular ingrowth may impair collagen turnover and extracellular matrix organization, making the graft vulnerable to mechanical fatigue under cyclic loading. This is particularly concerning in hamstring tendon grafts, where delayed revascularization and lower baseline mechanical strength may exacerbate the risk. The role of biological augmentation (e.g., platelet-rich plasma (PRP) or stem cells) in mitigating elongation remains an area for future exploration [64].

The identification of elongation as a separate and prevalent failure mode has meaningful clinical implications. This highlights the importance of incorporating serial objective laxity measurements during follow-up rather than relying solely on patient-reported outcomes or imaging findings. This recognition also supports the adoption of criteria-based rehabilitation protocols that emphasize strength and neuromuscular assessments over fixed timelines. Furthermore, reevaluation of surgical techniques—particularly tunnel placement and graft fixation—may be encouraged to minimize micromotion and early graft overload. As elongation can result in subtle functional deficits rather than overt graft rupture, it should be considered a potential cause of persistent instability or patient dissatisfaction, even when imaging demonstrates graft continuity.

### Limitations

Several limitations must be acknowledged. First, the small number of studies specifically addressing reconstructed ACL rupture localization (*n* = 3) limited the power of subgroup comparisons and increased susceptibility to publication bias. Second, the variability in definitions and detection methods for elongation (instrumented laxity versus clinical judgment) may have introduced measurement bias. Third, the included studies demonstrated substantial methodological and clinical heterogeneity—reflected by high *I*^2^ values across most analyses—which represents a clear source of bias and limits the precision and generalizability of the pooled estimates. Fourth, graft type, surgical technique, and fixation methods varied across studies, which may have confounded pooled estimates. Finally, in studies of double-bundle ACLR, the AM and PL bundles were analyzed as independent observations to preserve anatomical detail. While this method aligns with prior studies, it may introduce partial statistical non-independence and should therefore be interpreted with caution.

## Conclusions

This meta-analysis found no statistically significant differences in rupture-site localization between first ruptures and reruptures, indicating that reconstructed ACLs tend to fail in anatomical patterns resembling those of the native ligament. However, the markedly higher incidence of graft elongation observed in the rerupture cohort suggests a distinct mode of graft failure. Although factors such as tunnel position, graft type, or biological remodeling may contribute to this phenomenon, these associations remain hypothesis-generating and should be confirmed in future research.

From a clinical perspective, these findings highlight graft elongation as a unique mechanism of failure—distinct from traumatic rupture—that warrants dedicated preventive and rehabilitation strategies. Optimizing surgical technique and enhancing biological graft integration may help reduce the risk of elongation and improve long-term outcomes following ACL reconstruction.

## Supplementary Information

Supplementary material 1.

## Data Availability

Raw data are available upon request to the corresponding author.
