# Supplementary material for: Primary versus rerupture of the anterior cruciate ligament: rupture site patterns and graft elongation—a systematic review and meta-analysis
Source: Knee Surg Relat Res. 2026 Apr 3;38:15. doi: 10.1186/s43019-026-00313-9 (PMC13049893; doi:10.1186/s43019-026-00313-9)
Supplement: Supplementary file 1 — Supplementary material 1. [file 43019_2026_313_MOESM1_ESM.docx]

# Metanalisi: Distal (tibial)

Grafici: *Forest1* e *Funnel1 e Doi1*

**Number of analysed studies and patients**

Number of studies: 15

Number of analysed patients: 1533

Number of analysed patients with distal lesion: 103

## 2.1 Overall

|  | Estimate [95%CI] |
| --- | --- |
| Pooled percentage (Random effects model) | 4.87 [2.15; 8.44] |
| **Heterogeneity** |  |
| Tau^2^ | 0.0138 [0.0071; 0.0480] |
| I^2^ | 84.2% [75.3%; 89.9%] |

Test of heterogeneity: Q_14_= 88.66, p<0.001*

## By group

| **Characteristics of the model** | **Values** |
| --- | --- |
| Tau^2^ | 0.0152 (SE = 0.0087) |
| Tau | 0.1231 |
| R^2^ | 0.00% |
| **Test for subgroup differences** |  |
| Within group | Q_13_= 88.66, p-value<0.001* |
| Between group | Q_1_= 0.02, p-value=0.891 |

Estimates

|  | N of studies | Percentage [95%CI] | Q | I^2^ | Subgroup comparison  (p-value) |
| --- | --- | --- | --- | --- | --- |
| Total | 15 | 4.88 [2.08; 8.60] | 88.66 | 84.2% | - |
| **Subgroup** |  |  |  |  |  |
| First rupture | 11 | 4.76 [1.63; 9.12] | 83.98 | 88.1% | Reference group |
| Re-rupture | 4 | 5.24 [0.49; 13.44] | 4.68 | 35.9% | 0.891 |

## 2.3 Funnel plot & doi plot

| rank correlation test | p-value: 0.620 |
| --- | --- |
| linear regression test | p-value: 0.656 |
| Doi plot | no asymmetry |
| LFK index | 0.28 |


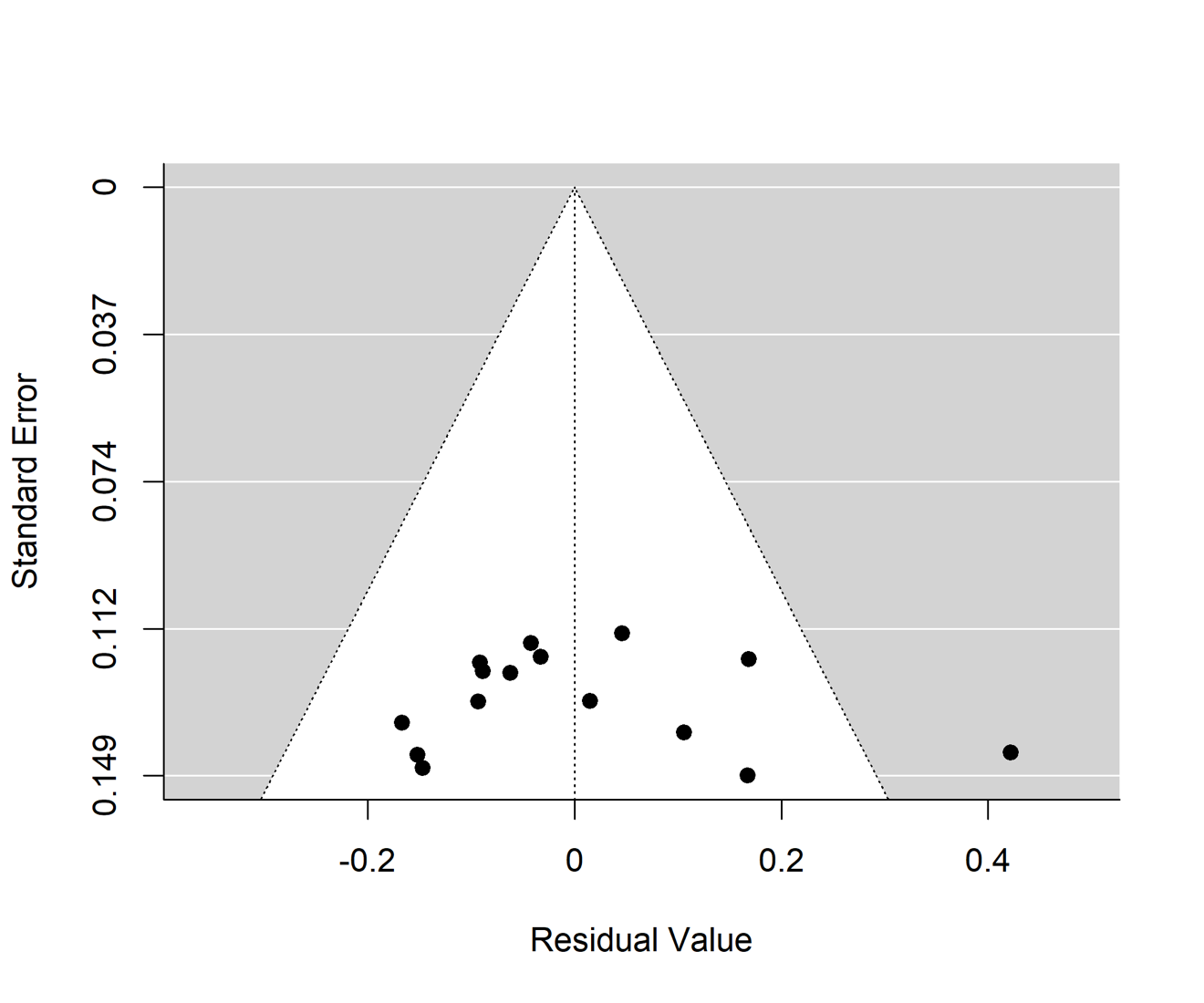


**
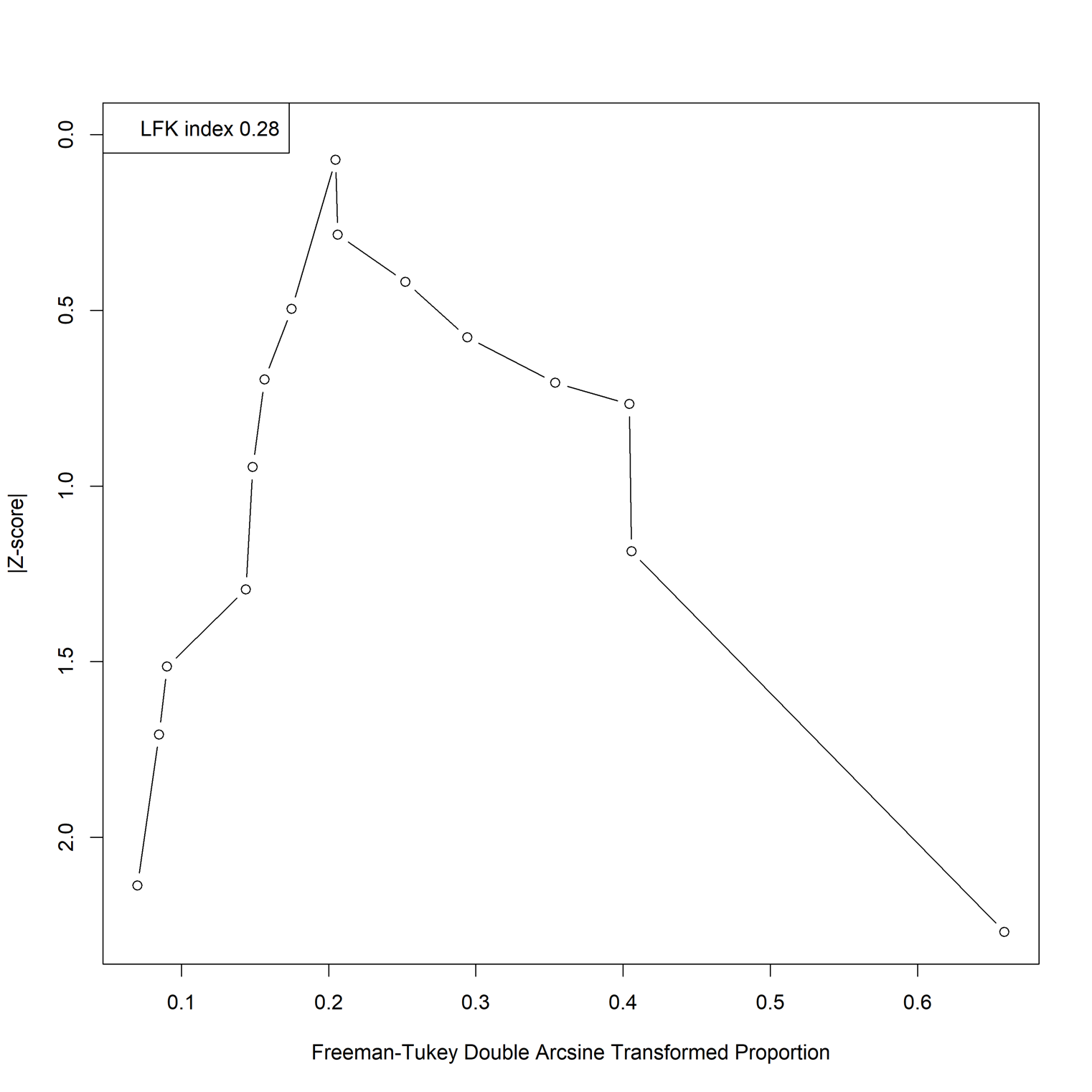
**

**Sensitivity analysis**

- Overall estimate with Trim-and-fill method

Number of studies added: 2

Number of studies: 17

|  | Estimate [95%CI] |
| --- | --- |
| Pooled percentage (random effects model) | 5.86 [2.96; 9.52] |
| **Heterogeneity** |  |
| tau^2^ | 0.0146 [0.0079; 0.0470] |
| I^2^ | 84.0% [75.7%; 89.5%] |

**Test of heterogeneity:** Q_16_= 100.15, p<0.001*

- Fail-safe N Calculation Using the Rosenthal Approach

Fail-safe N: 1654

# Metanalisi: Mid-substance

Grafici: *Forest2* e *Funnel2 e Doi2*

**Number of analysed studies and patients**

Number of studies: 15

Number of analysed patients: 1533

Number of analysed patients with distal lesion: 786

## 3.1 Overall

|  | Estimate [95%CI] |
| --- | --- |
| Pooled percentage (Random effects model) | 42.47 [31.45; 53.86] |
| **Heterogeneity** |  |
| Tau^2^ | 0.0450 [0.0294; 0.1620] |
| I^2^ | 94.5% [92.4%; 96.1%] |

Test of heterogeneity: Q_14_= 256.83, p<0.001*

## By group

| **Characteristics of the model** | **Values** |
| --- | --- |
| Tau^2^ | 0.0469 (SE = 0.0252) |
| Tau | 0.2165 |
| R^2^ | 0.0% |
| **Test for subgroup differences** |  |
| Within group | Q_13_= 246.96, p-value<0.001* |
| Between group | Q_1_= 0.50, p-value=0.481 |

Estimates

|  | N of studies | Percentage [95%CI] | Q | I^2^ | Subgroup comparison  (p-value) |
| --- | --- | --- | --- | --- | --- |
| Total | 15 | 42.44 [31.21; 54.05] | 256.83 | 92.4% | - |
| **Subgroup** |  |  |  |  |  |
| First rupture | 11 | 44.94 [31.76; 58.48] | 208.01 | 95.2% | Reference group |
| Re-rupture | 4 | 35.63 [16.03; 58.02] | 38.95 | 92.3% | 0.481 |

## 3.3 Funnel plot & doi plot

| rank correlation test | p-value: 0.075 |
| --- | --- |
| linear regression test | p-value: 0.019* |
| Doi plot | major asymmetry |
| LFK index | -2.08 |


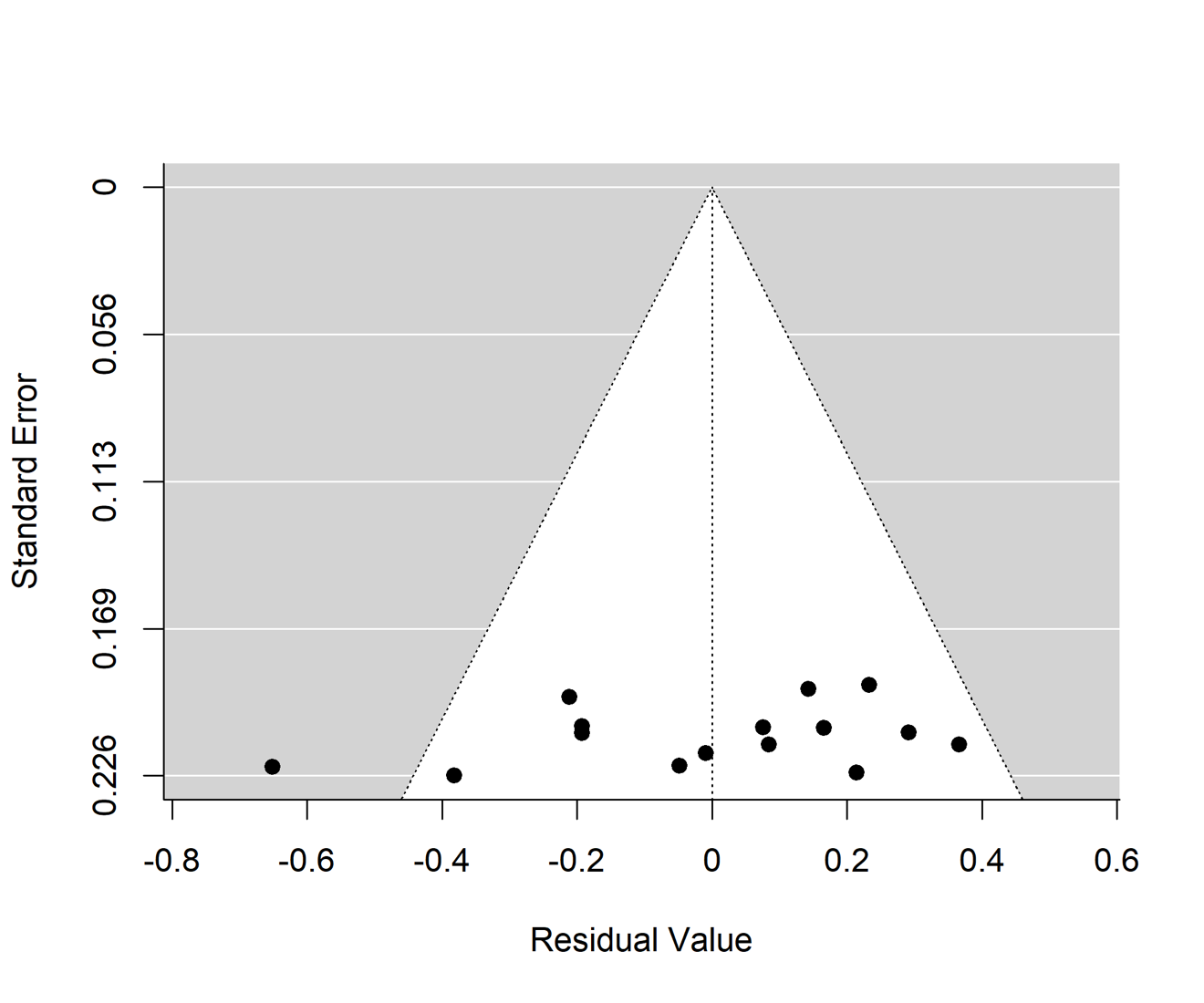


**
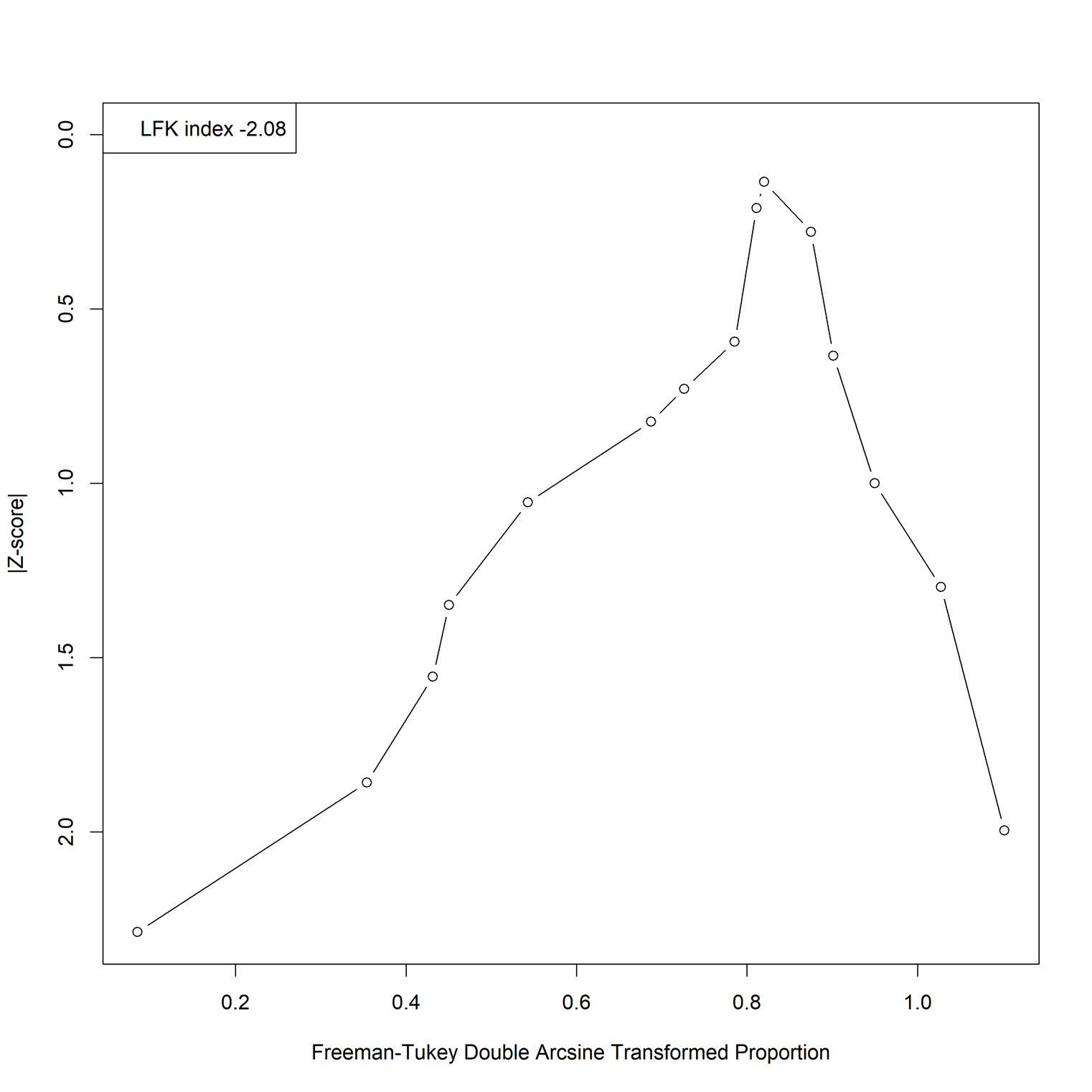
**

**Sensitivity analysis**

- Overall estimate with Trim-and-fill method

Number of studies added: 4

Number of studies: 19

|  | Estimate [95%CI] |
| --- | --- |
| Pooled percentage (random effects model) | 55.56 [43.13; 67.66] |
| **Heterogeneity** |  |
| tau^2^ | 0.0688 [0.0533; 0.2346] |
| I^2^ | 95.9% [94.6%; 96.8%] |

**Test of heterogeneity:** Q_18_= 435.08, p<0.001*

- Fail-safe N Calculation Using the Rosenthal Approach

Fail-safe N: 16228

# Metanalisi: Proximal (femoral)

Grafici: *Forest3* e *Funnel3 e Doi3*

**Number of analysed studies and patients**

Number of studies: 15

Number of analysed patients: 1533

Number of analysed patients with distal lesion: 572

## 4.1 Overall

|  | Estimate [95%CI] |
| --- | --- |
| Pooled percentage (Random effects model) | 42.04 [30.55; 53.98] |
| **Heterogeneity** |  |
| Tau^2^ | 0.0497 [0.0314; 0.1724] |
| I^2^ | 95.0% [93.2%; 96.4%] |

Test of heterogeneity: Q_14_= 282.6, p<0.001*

## By group

| **Characteristics of the model** | **Values** |
| --- | --- |
| Tau^2^ | 0.0492 (SE = 0.0264) |
| Tau | 0.2217 |
| R^2^ | 1.13% |
| **Test for subgroup differences** |  |
| Within group | Q_13_= 258.48, p-value<0.001* |
| Between group | Q_1_= 1.89, p-value=0.170 |

Estimates

|  | N of studies | Percentage [95%CI] | Q | I^2^ | Subgroup comparison  (p-value) |
| --- | --- | --- | --- | --- | --- |
| Total | 15 | 42.04 [30.60; 53.91] | 282.62 | 95.0% | - |
| **Subgroup** |  |  |  |  |  |
| First rupture | 11 | 47.02 [33.44; 60.83] | 243.79 | 95.9% | Reference group |
| Re-rupture | 4 | 28.78 [10.63; 51.24] | 14.69 | 79.6% | 0.170 |

## 4.3 Funnel plot & doi plot

| rank correlation test | p-value: 0.048* |
| --- | --- |
| linear regression test | p-value: 0.385 |
| Doi plot | minor asymmetry |
| LFK index | 1.34 |


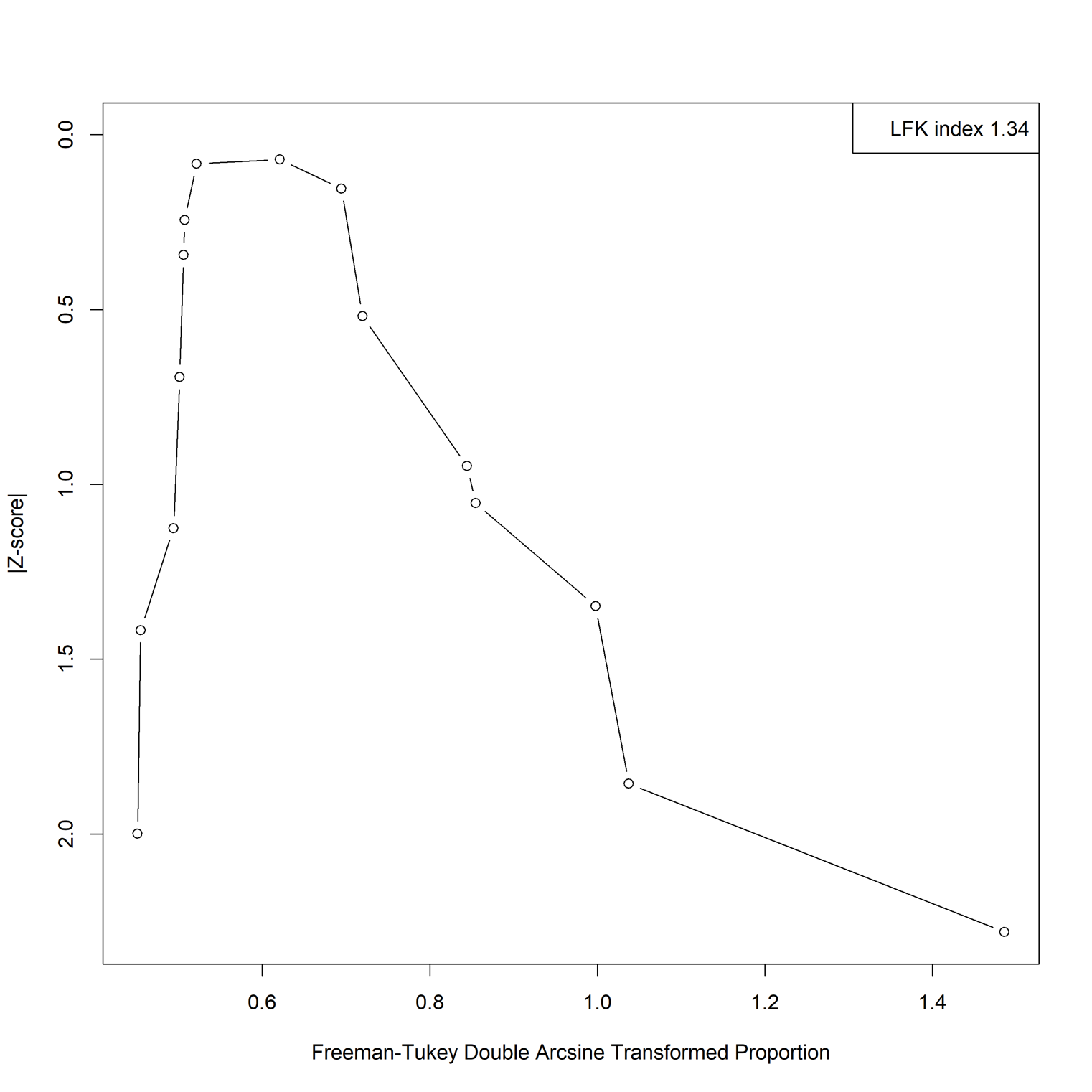

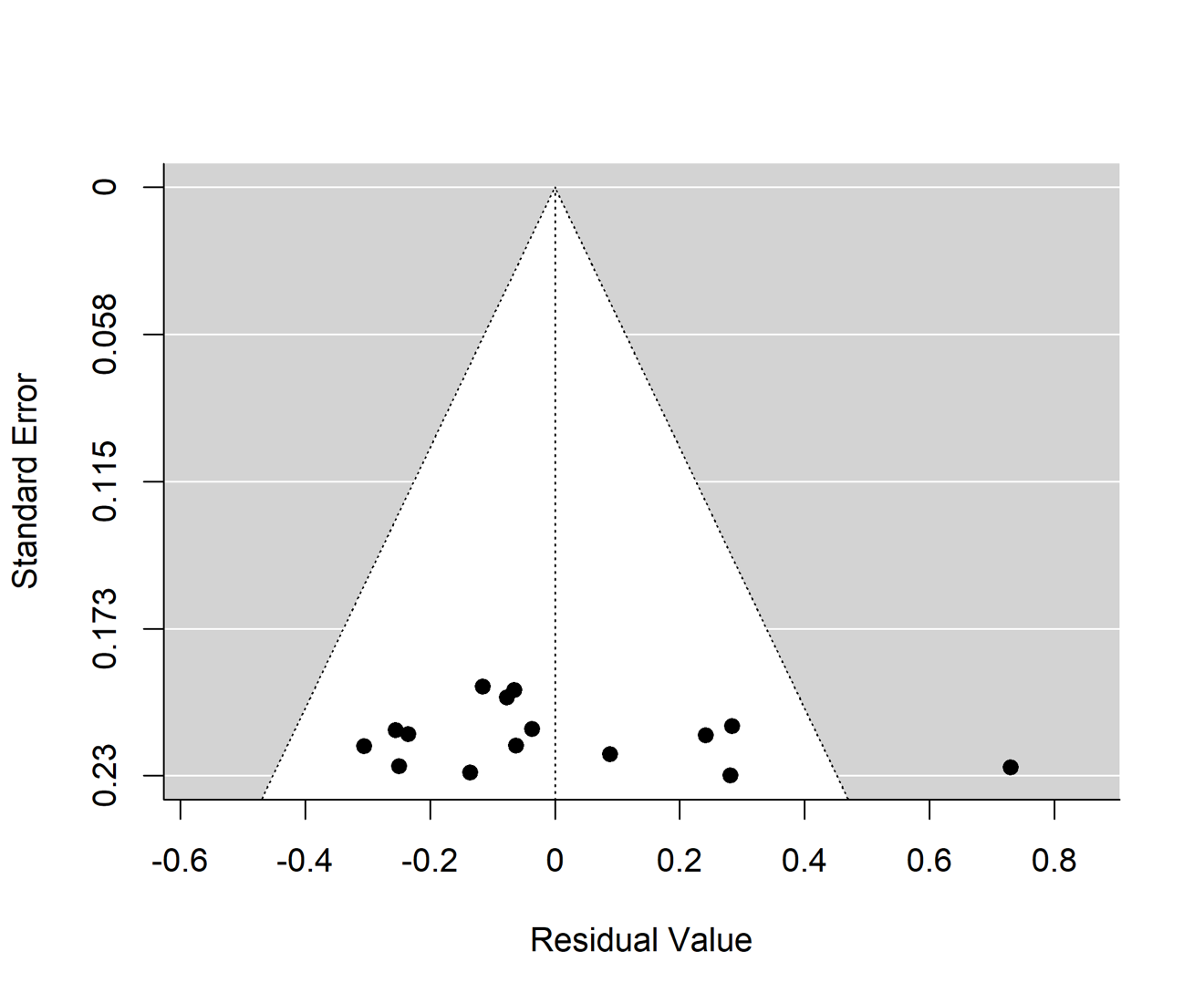


**Sensitivity analysis**

- Overall estimate with Trim-and-fill method

Number of studies added: 1

Number of studies: 16

|  | Estimate [95%CI] |
| --- | --- |
| Pooled percentage (random effects model) | 36.67 [24.43; 49.81] |
| **Heterogeneity** |  |
| tau^2^ | 0.0665 [0.0476; 0.2416] |
| I^2^ | 96.1% [94.8%; 97.1%] |

**Test of heterogeneity:** Q_15_= 384.15, p<0.001*

- Fail-safe N Calculation Using the Rosenthal Approach

Fail-safe N: 13226

# Metanalisi: Elongation

Grafici: *Forest4* e *Funnel4 e Doi4*

**Number of analysed studies and patients**

Number of studies: 15

Number of analysed patients: 1533

Number of analysed patients with distal lesion: 72

## 5.1 Overall

|  | Estimate [95%CI] |
| --- | --- |
| Pooled percentage (Random effects model) | 2.75 [0.04; 8.08] |
| **Heterogeneity** |  |
| Tau^2^ | 0.0448 [0.0257; 0.1432] |
| I^2^ | 94.5% [92.4%; 96.1%] |

Test of heterogeneity: Q_14_= 282.6, p<0.001*

## By group

| **Characteristics of the model** | **Values** |
| --- | --- |
| Tau^2^ | 0.0056 (SE = 0.0037) |
| Tau | 0.0745 |
| R^2^ | 87.62% |
| **Test for subgroup differences** |  |
| Within group | Q_13_= 40.72, p-value<0.001* |
| Between group | Q_1_= 174.25, p-value<0.001* |

Estimates

|  | N of studies | Percentage [95%CI] | Q | I^2^ | Subgroup comparison  (p-value) |
| --- | --- | --- | --- | --- | --- |
| Total | 15 | 2.41 [0.95; 4.36] | 256.22 | 94.5% | - |
| **Subgroup** |  |  |  |  |  |
| First rupture | 11 | 0.00 [0.00; 0.52] | 1.94 | 0.0% | Reference group |
| Re-rupture | 4 | 26.51 [18.29; 35.60] | 38.78 | 92.3% | <0.001* |

## 5.3 Funnel plot & doi plot

| rank correlation test | p-value: 0.003* |
| --- | --- |
| linear regression test | p-value: 0.422 |
| Doi plot | major asymmetry |
| LFK index | 2.94 |


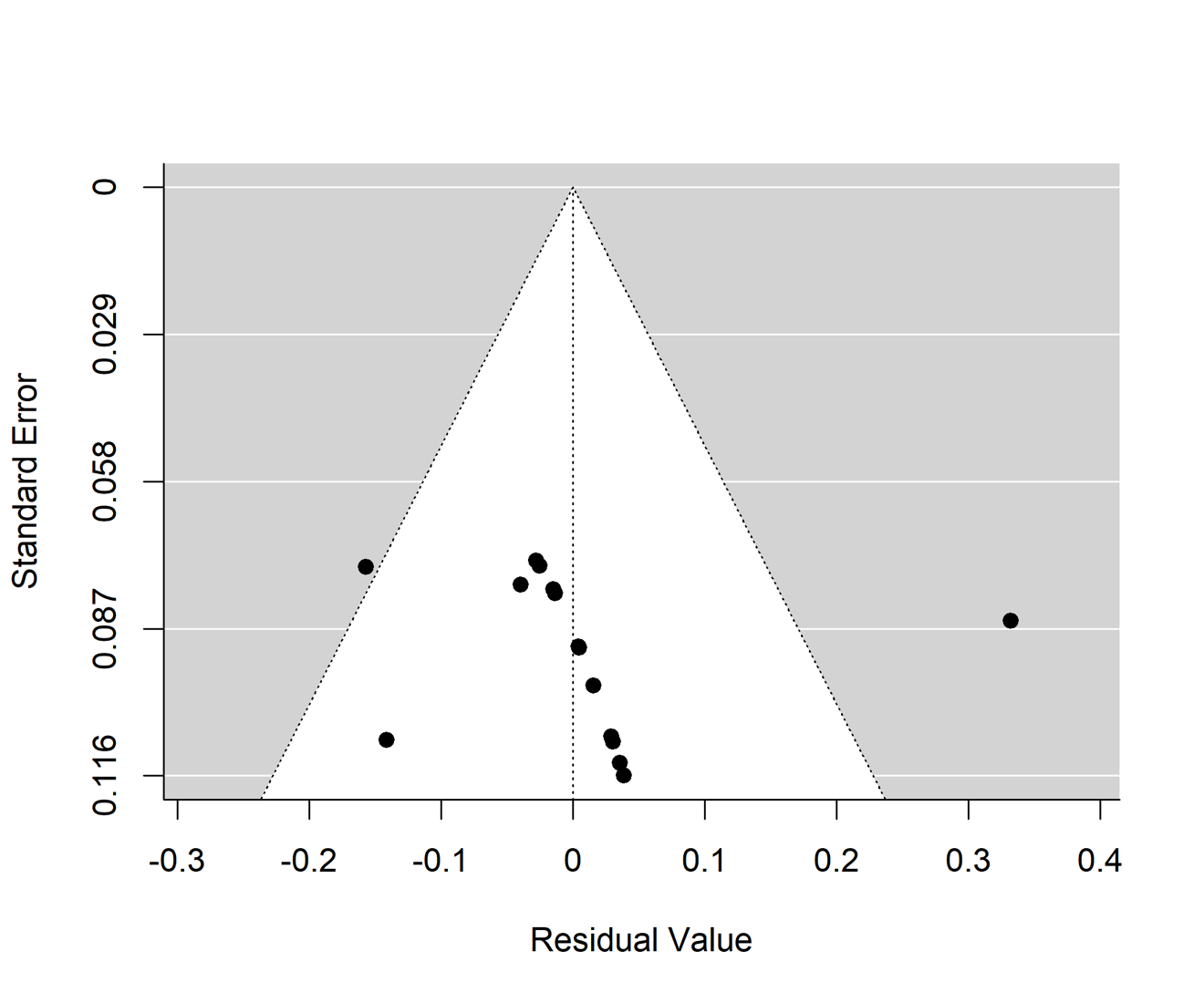


**
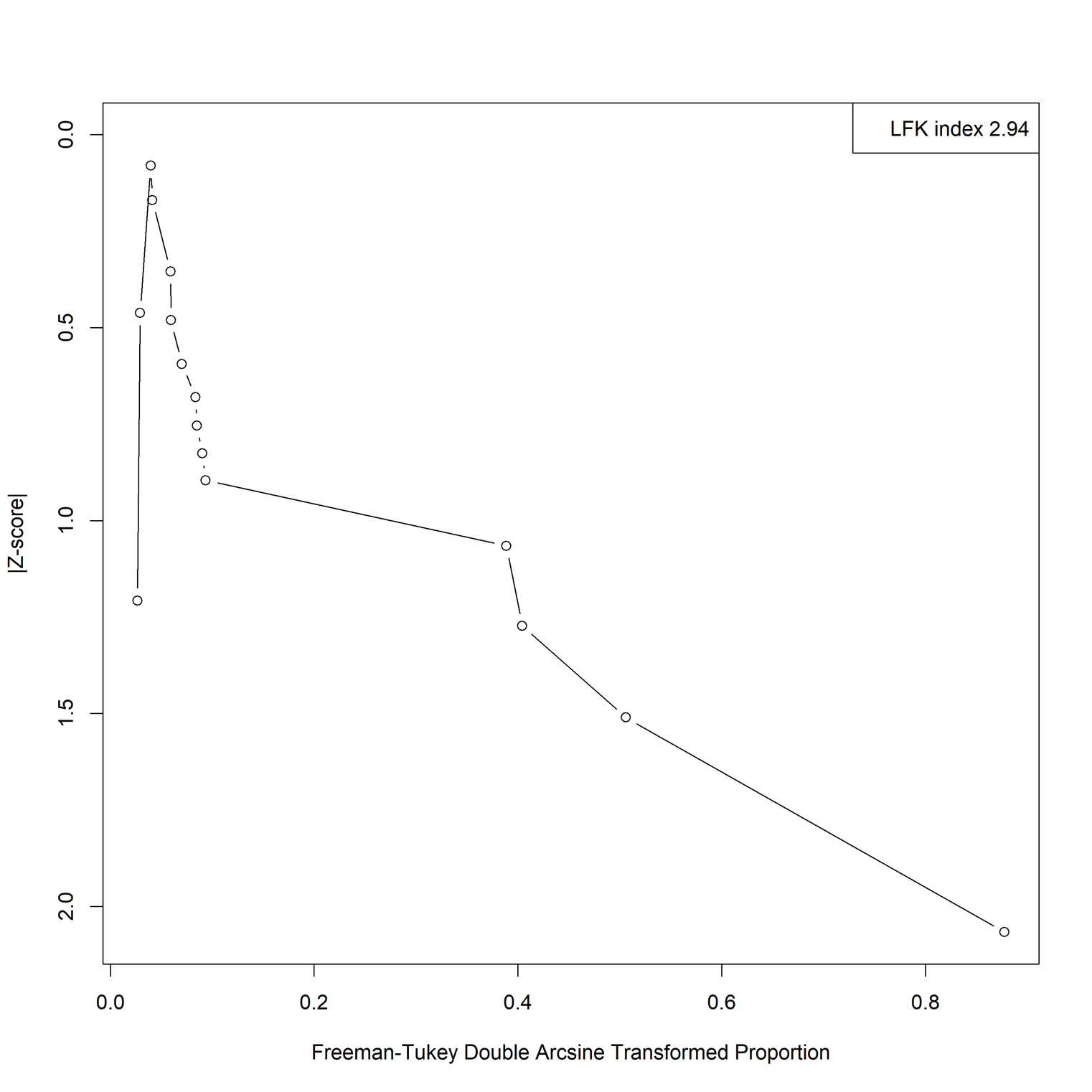
Sensitivity analysis**

- Overall estimate with Trim-and-fill method

Number of studies added: 0

Number of studies: 15

|  | Estimate [95%CI] |
| --- | --- |
| Pooled percentage (random effects model) | 2.75 [0.04; 8.08] |
| **Heterogeneity** |  |
| tau^2^ | 0.0448 [0.0257; 0.1432] |
| I^2^ | 94.5% [92.4%; 96.1%] |

**Test of heterogeneity:** Q_14_= 256.22, p<0.001*

- Fail-safe N Calculation Using the Rosenthal Approach

Fail-safe N: 762
